# Design, Analysis and Testing of a Novel Mitral Valve for Transcatheter Implantation

**DOI:** 10.1007/s10439-017-1828-2

**Published:** 2017-04-03

**Authors:** Selim Bozkurt, Georgia L. Preston-Maher, Ryo Torii, Gaetano Burriesci

**Affiliations:** 1 UCL Mechanical Engineering, Cardiovascular Engineering Laboratory, University College London, London, WC1E 7JE UK; 2 Ri.MED Foundation, Bioengineering Group, Palermo, Italy

**Keywords:** Transcatheter mitral valve implantation (TMVI), Heart valve development, Heart valve assessment, Mitral valve, Bioprosthetic bi-leaflet valve

## Abstract

Mitral regurgitation is a common mitral valve dysfunction which may lead to heart failure. Because of the rapid aging of the population, conventional surgical repair and replacement of the pathological valve are often unsuitable for about half of symptomatic patients, who are judged high-risk. Transcatheter valve implantation could represent an effective solution. However, currently available aortic valve devices are inapt for the mitral position. This paper presents the design, development and hydrodynamic assessment of a novel bi-leaflet mitral valve suitable for transcatheter implantation. The device consists of two leaflets and a sealing component made from bovine pericardium, supported by a self-expanding wireframe made from superelastic NiTi alloy. A parametric design procedure based on numerical simulations was implemented to identify design parameters providing acceptable stress levels and maximum coaptation area for the leaflets. The wireframe was designed to host the leaflets and was optimised numerically to minimise the stresses for crimping in an 8 mm sheath for percutaneous delivery. Prototypes were built and their hydrodynamic performances were tested on a cardiac pulse duplicator, in compliance with the ISO5840-3:2013 standard. The numerical results and hydrodynamic tests show the feasibility of the device to be adopted as a transcatheter valve implant for treating mitral regurgitation.

**Electronic supplementary material:**

The online version of this article (doi:10.1007/s10439-017-1828-2) contains supplementary material, which is available to authorized users.

## Introduction

Mitral regurgitation is one of the major mitral valve pathologies leading to heart failure.^27^ It is a result of primary anatomical changes affecting the mitral valve leaflets, or left ventricular remodelling which may lead to dislocation of papillary muscles.^15^ Although mild and moderate mitral regurgitation may be tolerated and do not require surgical intervention, patients with severe symptomatic mitral regurgitation have a very low survival rate in absence of interventions^40^ which restore the coaptation of the mitral valve leaflets,^11^ or replace the mitral valve with a prosthetic device.^30^ While non-randomised reports suggest that repairing techniques have significantly lower mortality rates,^54^ randomised studies indicate no significant difference in the mortality rates^3^ between replacement and repair^20^ in ischemic related mitral regurgitation. Whenever practicable, surgical repair remains the best option for the treatment of degenerative mitral regurgitation.^19^,^20^ Nevertheless, in elderly patients surgical intervention is often associated with comorbidities such as diabetes, pulmonary disease, perioperative hemodialysis and low ejection fraction, which increase considerably the risk of operative mortality.^5^,^49^ As a result, only a small portion of patients suffering from functional mitral regurgitation and approximately half of those suffering from degenerative mitral regurgitation currently undergo surgery.^7^ Minimally invasive transcatheter implantation can reduce the risks in these patients and offer an alternative to surgical therapies for mitral valve diseases.^34^

Transcatheter techniques to treat mitral regurgitation can be classified as leaflet and chordae repair; indirect annuloplasty; left ventricular remodelling; and replacement.^25^ Leaflet and chordae repair techniques can be effective and durable in a wide variety of pathologies, even without annuloplasty in selected patients.^21^,^36^ Indirect annuloplasty releases devices which support remodelling of the annulus in the coronary sinus, improving leaflet coaptation. However this procedure is associated with adverse cardiovascular events, such as myocardial infarction and coronary sinus rupture,^24^,^47^ and data available on the short- and long-term outcome are still limited.^32^,^37^ Left ventricular remodelling is applied to reduce a dilated left ventricle diameter which may tether the mitral valve leaflets.^22^ Despite the initial attempts demonstrated benefits, this technique is not available commercially at the moment.

Although these transcatheter techniques can successfully reduce mitral regurgitation, a valve replacement would allow to restore the unidirectional blood flow in a wider patients’ anatomical selection. Transcatheter mitral valve (TMV) replacements, which attempt to conjugate the lessons from surgical mitral valve interventions^35^,^42^ with the successful transcatheter aortic valve (TAV) experience, are still in developmental stages. A number of TMVs have been proposed, and are at different stages of evaluation.^1^,^23^,^41^ These are typically adapted from TAVs,^41^ and adopt the same three leaflets circular configuration. Possible issues that may arise with these devices include suboptimal placement in native mitral position, due to the irregular non-circular shape of the mitral annulus, and recurrence of paravalvular leakage.^30^ This is known to reduce the survival rates after TAV replacement, and is a more critical problem for mitral valve implants, where the implantation sizes and the peak transvalvular pressures are higher.^25^

In this paper, a novel mitral valve device suitable for transcatheter implantation, based on a bi-leaflet configuration with D-shaped orifice, is presented. In particular, the development of the proposed valve, in terms of design optimisation and *in vivo* hydrodynamic assessment is described.

## Materials and Methods

### Leaflet Design Optimisation and Manufacturing

Leaflets were designed to minimise structural and functional failure. Structural failure typically occurs due to excessive stresses, with the locations of structural failure in explanted bioprosthetic heart valves often associated with the peak regions of maximum principal stress.^9^ Design optimisation was performed using parametrically-varied CAD models by means of finite element analysis for both structural and functional criteria.

**Figure 1:**
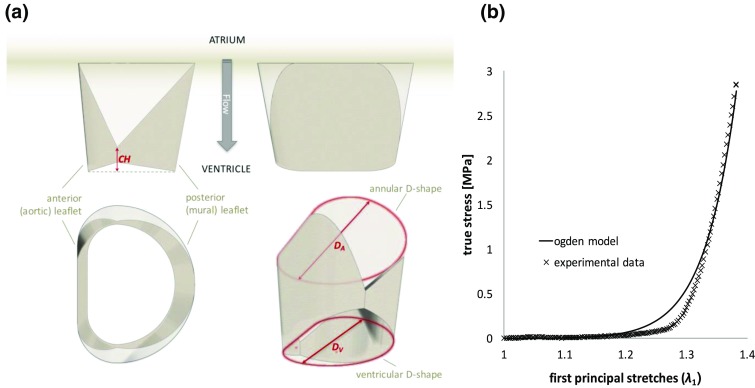
(a) Sketch of the leaflets design: *CH* represents the coaptation height, *D*_*V*_ and *D*_*A*_ are the dimensions used to define scale factor (*SF*) in the design. (b) Experimental data points describing the constitutive behavior of the used pericardium, and fitted curve with the adopted Ogden model.

Leaflets were designed to lie, in their unstressed open configuration, on a ruled surface characterised by a D-shaped orifice cross section with a ratio between the antero-posterior and the inter-commissural diameters equal to 3:4 (Fig. 1). Similarly to healthy native mitral valve,^58^ leaflets were designed with a conical shape, reducing their cross section linearly form the inlet to the outlet. This solution was preferred to minimise the risk of ventricular outflow tract obstruction, by decreasing the tendency of the leaflets to diverge from their design configuration, especially when the valve is placed in annuli significantly smaller than the nominal valve dimension. Also, shorter free edges were observed to reduce the leaflets fluttering during diastole, which is typically associated with increased calcification, haemolysis, regurgitation and early fatigue failure.^6^ A scale factor (*SF*), defined as the ratio between the outlet (*D*_*V*_) and inlet (*D*_*A*_) intertrigonal dimensions of the device (Fig. 1a), was introduced to quantify the leaflets conicity in the free unloaded configuration. A set of five scale factors of 0.745, 0.798, 0.852, 0.906 and 0.960 were chosen for investigation, with the smallest corresponding to a maximum reduction of the D-shape cross sectional area from the base to the edge of the leaflets equal to 60%. A coaptation height parameter, *CH*, was defined, referring to the vertical distance from the arris between the aortic and mural leaflets to the middle of the leaflets free edge. This has the function to allow the adjustment of the leaflets edge and avoid excess of redundant material, which results in localised buckling, commonly associated with failure of pericardial leaflets.^50^ Five evenly spaced coaptation lengths were chosen for investigation, from 0 to 30% of the leaflets height. The combination of five scale factors and coaptation lengths resulted in twenty-five incrementally different bi-leaflet CAD models.

The leaflets were designed in their assembled configuration as surfaces using 3D CAD software Rhinoceros 4.0 (Robert McNeel & Associates), using an inter-trigonal dimension equal to 26 mm. Numerical analyses of structural mechanics were performed using an explicit solver in LS-DYNA (Livermore Software Technology Corporation). The analysis of the twenty-five initial designs provided coaptation area and peak maximum principal stress data for hypertensive systolic loading conditions, i.e. when they are fully closed and a peak of transmitral pressure equal to 200 mmHg is applied.

Glutaraldehyde fixed bovine pericardium was selected as material for the leaflets, due to its long clinical use in bioprosthetic heart valves and favorable hemodynamic performance.^26^ Calf pericardial sacs were obtained from a local abattoir, and fixed in a 0.5% solution of glutaraldehyde for 48 h, after removing the fat and parietal pericardium by hand.^26^ Three sets of leaflets were obtained from visually homogeneous regions of the pericardial sac of thickness in the range of 400 *μ*m ±10% (measured using a thickness gauge - Mitutoyo Corporation, Tokyo, Japan). One dumbbell-shaped sample of 4 mm width and 16 mm gauge length was extracted from the unused portion of each patch, using a die cutter.

Specimens were conditioned with uniaxial tensile cycles from 0 to 6 N with 20 mm/min rate until stabilisation, using a ZwickiLine testing machine (Zwick/Roell, Germany) equipped with a media container maintaining 40 °C, and used to determine the representative mechanical properties for the used material. The constitutive behaviour observed for the treated pericardium was modeled in the numerical analyses using a four parameters Ogden equation:

1 $$W = \frac{{\mu_{1} }}{{\alpha_{1} }}\left( {\lambda_{1}^{{\alpha_{1} }} + \lambda_{2}^{{\alpha_{1} }} + \lambda_{3}^{{\alpha_{1} }} - 3} \right) + \frac{{\mu_{2} }}{{\alpha_{2} }}\left( {\lambda_{1}^{{\alpha_{2} }} + \lambda_{2}^{{\alpha_{2} }} + \lambda_{3}^{{\alpha_{2} }} - 3} \right)$$

where the strain energy density *W* is expressed in terms of the principal stretches *λ*_1_, *λ*_2_ and *λ*_3_, and the four material constants *μ*_1_, *μ*_2_, *α*_1_ and *α*_2_. The material constants best fitting the average stress–strain curve obtained from the experiments were: *μ*_1_ = 7.6 × 10^−6^; *μ*_2_ = 5.7 × 10^−4^; *α*_1_ = *α*_2_ = 26.26 (*R* ^2^ = 0.981). The experimental data points and fitted curve are reported in the graph in Fig. 1b.

The coaptation of the leaflets was modelled using a frictionless master-slave contact condition.^9^ The effect of the inertia of blood in reducing system oscillations was reproduced by using a damping coefficient of 0.9965, consistent with what identified in previous works based on similar simulations.^9^ Each leaflet was discretised with approximately 1820 quadrilateral 2D constant strain Belytschko-Lin-Tsay shell elements with 5 points of integration across the thickness. The leaflet thickness was set to 0.4 mm, approximating the value selected for the patches used for the valve manufacturing. To simulate leaflet closure, a uniformly distributed opening pressure of 4 mmHg was initially applied to the leaflets, starting from their unloaded position, and then reverted and ramped to a closing pressure of 115 mmHg. This corresponds to the typical mean transmitral systolic pressure difference obtained by testing the valve prototypes in the pulse duplicator, for a cardiac output of 5 L/min, a frequency of 70 beats per minute (with 65% of diastolic time) and a normotensive aortic pressure of 100 mmHg. A minimum safety factor of 3, based on the strength reported for glutaraldehyde fixed bovine pericardial tissue,^4^ was accepted for the predicted peaks of stress.

### Frame Design and Optimisation

The TMV frame is designed to match and support the two leaflets along their constrained edge and provide their anchoring. Its structure is obtained from super elastic NiTi wires of 0.6 mm diameter.

**Figure 2:**
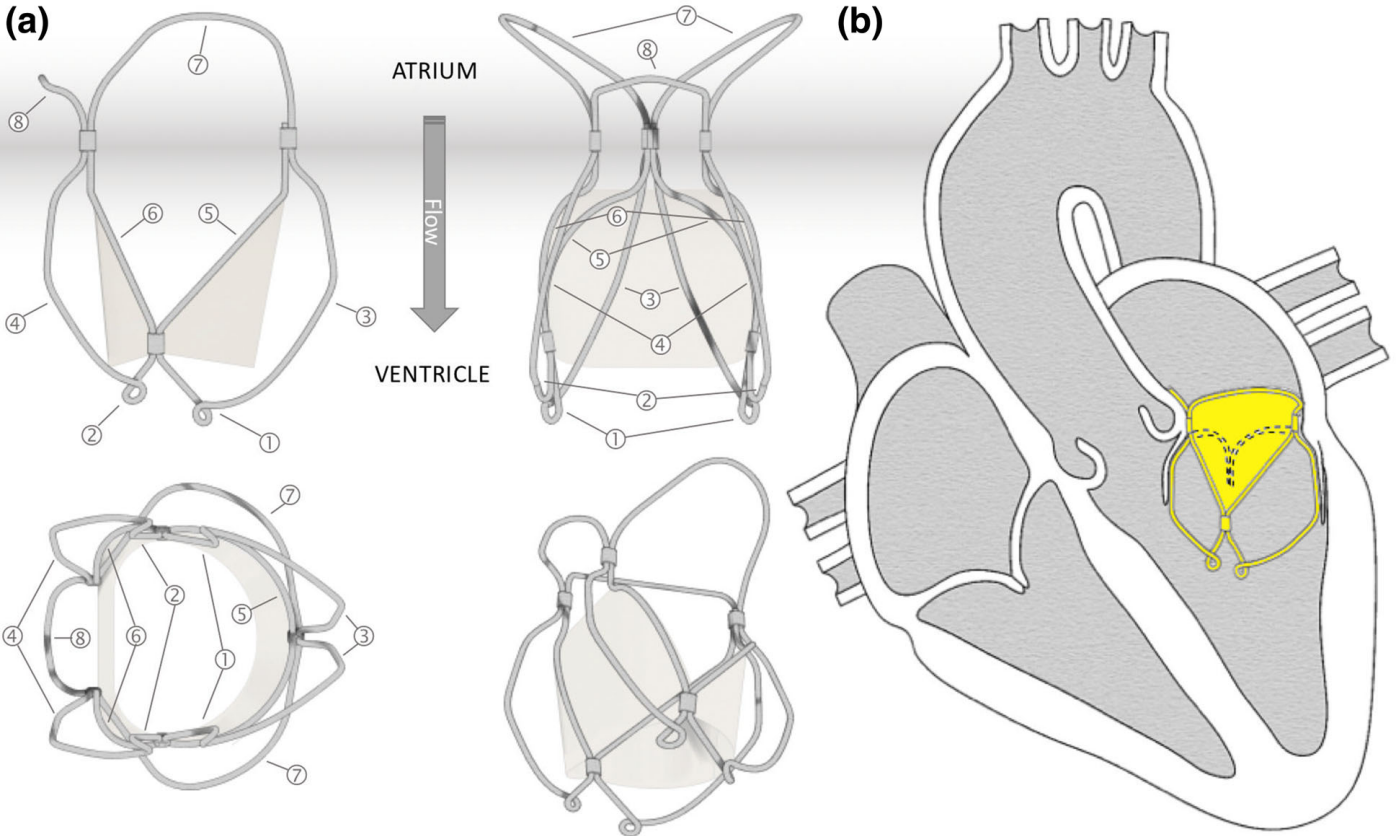
(a) Sketch of the valve wireframe; and (b) schematic representation of the implanted prosthetic valve.

The valve anchoring to the host anatomy is provided by the counteracting action from a set of proximal smoothly arched ribs, expanding into the atrium (portions 7 and 8 in Fig. 2a) and two petal-like structures protruding into the ventricle between the native mitral leaflets (portions 3 and 4 in Fig. 2a). The portion of the petals engaging with the anterior native leaflets (portions 4 in Fig. 2a) are designed to keep this in tension by expanding its anterioro-lateral and posterior-medial parts^12^ laterally, in the attempt to reduce its systolic motion without pushing it markedly in subaortic position and minimise the risk of left ventricular outflow tract obstruction.^59^

The distal margin of the ventricular structures includes distal loops (portions 1 and 2 in Fig. 2) which act as torsion springs, reducing the levels of stress in the crimped frame and dampening the load experienced by the leaflets during the operating cycles. The loops are also used to host control tethers which allow the valve recollapse into a delivery sheath by adopting the same approach described in Rahmani *et al.* 45

3D solid models of the wireframe (Fig. 2) were developed using NX CAD (Siemens PLM Software) program. Each solid model was discretised with approximately 110,000 tetrahedral elements of maximum edge size equal to 0.2 mm. The wireframe was modeled as NiTi shape memory alloy by using an austenitic Young’s modulus (*E*_A_) of 50 GPa, martensitic Young’s modulus (*E*_M_) of 25 GPa, and 0.3 for the Poisson’s ratio of both austenitic and martensitic (*ν*_A_ *, ν*_M_) phases.^56^ The transformation stresses of the NiTi wire for the austenite start (*σ*_as,s_), austenite finish (*σ*_as,f_), martensite start (*σ*_sa,s_) and martensite finish (*σ*_sa,f_) were 380, 400, 250 and 220 MPa respectively.^56^ The sleeves were modeled as stainless steel by using a Young’s modulus of 210 kN/mm^2^ and a Poisson’s ratio of 0.3, and were connected to the wireframe by applying stress free projected glued contact to their surfaces. The relative motion between the TMV and catheter during crimping was simulated by fixing the displacement of the top of the loops.

The wireframe geometry was optimised to maintain the maximum von Mises stress below the martensitic yield stress, when crimped to 8 mm (24 French) diameter. Simulations were performed using the FEA software MSC.Marc/Mentat and an implicit solver utilizing single-step Houbolt time integration algorithm, by gradually reducing the diameter of a surround cylindrical contact surface. Critical regions subjected to the highest levels of stress during crimping were identified in the initial geometry and optimised iteratively, using the approach described in Burriesci *et al.* 10 For each portion indicated in Fig. 2, the length, curvature and angle values were updated in each simulation in order to obtain a parameter set minimising the crimping stress on the wireframe.

### Valve Prototypes

Prototypes of the wireframe structure were manufactured by thermomechanical processing of nitinol wires, mechanically joined at specific locations by means of stainless steel crimping sleeves. The leaflets and the sealing cuff made from bovine pericardium were sutured to the inner portions of the frame extensions (portions 5 and 6 in Fig. 2) using polypropylene surgical sutures. The skirt, made from a polyester mesh (Surgical Mesh PETKM2004, Textile Development Associates, USA), was included to gently distribute the anchoring force over the annulus (between portions 5, 6 and 7 in Fig. 2). The nominal valve size of the prototypes, defined based on the inter-trigonal dimension of the designed leaflets, was equal to 26 mm. This is suitable for preclinical *in vivo* evaluation in large animal models.

### Hydrodynamic Tests

**Figure 3:**
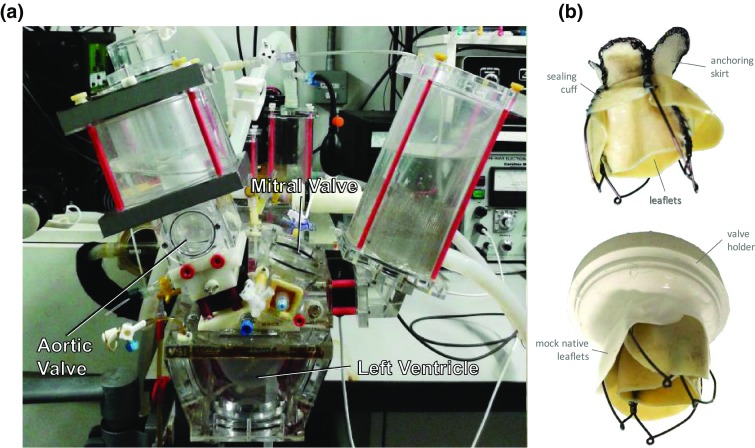
Experimental set-up for the hydrodynamic assessment of the proposed device: (a) pulse duplicator; (b) picture of the valve prototype indicating the leaflets, the sealing cuff and the anchoring skirt (top); and picture of the device after positioning in the valve holder (bottom).

The hydrodynamic performances of the three valve prototypes were assessed on a hydro-mechanical cardiovascular pulse duplicator system (*ViVitro Superpump SP3891, ViVitro, BC*) (Fig. 3). The flow through the heart valves is measured with two electromagnetic flow probes and two Carolina Medical flow meters (Carolina Medical Electronics, USA), and the pressures in the aorta, left ventricle and left atrium are acquired using Millar Mikro-Cath pressure transducers. The working fluid was buffers phosphate saline solution at 37 °C. Hydrodynamic assessment of the prototypes was performed at 70 bpm heart rate, 5 L/min mean cardiac output and 100 mmHg mean aortic pressure, in compliance with the ISO 5840-3:2013 standard. The pulse duplicator was operated to simulate systole/diastole ratio as 35/65 over a cardiac cycle and a bileaflet mechanical heart valve Sorin Bicarbon size 25 was used to represent the aortic valve. Silicone models of the mitral annulus and native leaflets were built, based on the geometry previously described in Lau *et al*.^33^ with inter-trigonal diameters ranging from 21 to 25 mm, and used to house the test valves. This dimensional range, at least one millimeter smaller than the nominal size of the test valve, was selected to allow some anchoring force and verify the valve securing and hydrodynamic performance over a large anatomical range.

Hydrodynamic performances of the prototypes were assessed by calculating the effective orifice area (EOA), regurgitant fraction and mean transmitral diastolic pressure. The effective orifice area was estimated using the Gorlin Equation (Eq. 2), as described in the ISO 5840.

2 $${\text{EOA}} = \frac{{Q_{\text{mv,rms}} }}{{51.6\sqrt {\Delta p_{\text{mv}} /\rho } }}$$

where, *Q*_mv,rms_ represents the root mean square of the flow rate through the mitral valve, Δ*p*_mv_ is the mean positive differential pressure across the mitral valve and *ρ* is the density of the circulating fluid. The regurgitant fraction is calculated as the ratio of the measured closing regurgitant volume (back flow during valve closure) plus the leakage volume (leaking flow after closure) and the forward flow volume during the ventricular filling.

## Results

**Figure 4:**
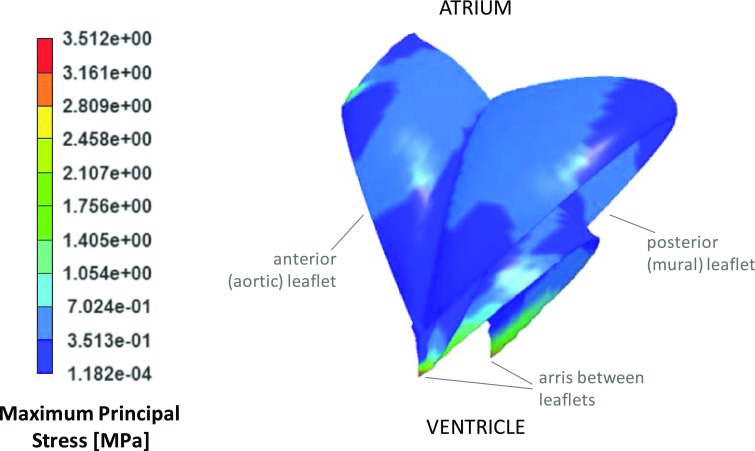
Maximum principal stress distribution for the optimal transcatheter mitral valve leaflets in their critical loading mode when fully closed, peak value 3.51 N/mm^2^ at the arris between the leaflets.

Seventeen of the twenty five bi-leaflet designs simulated numerically were functionally patent, and all had an acceptable peak of maximum principal stress below 5 MPa.^61^ Due to the need to ensure adequate valve function for a wide range of possible expansion sizes and shapes, the design providing maximum coaptation area was selected (Fig. 4) and the wireframe was subsequently made to fit this.

**Figure 5:**
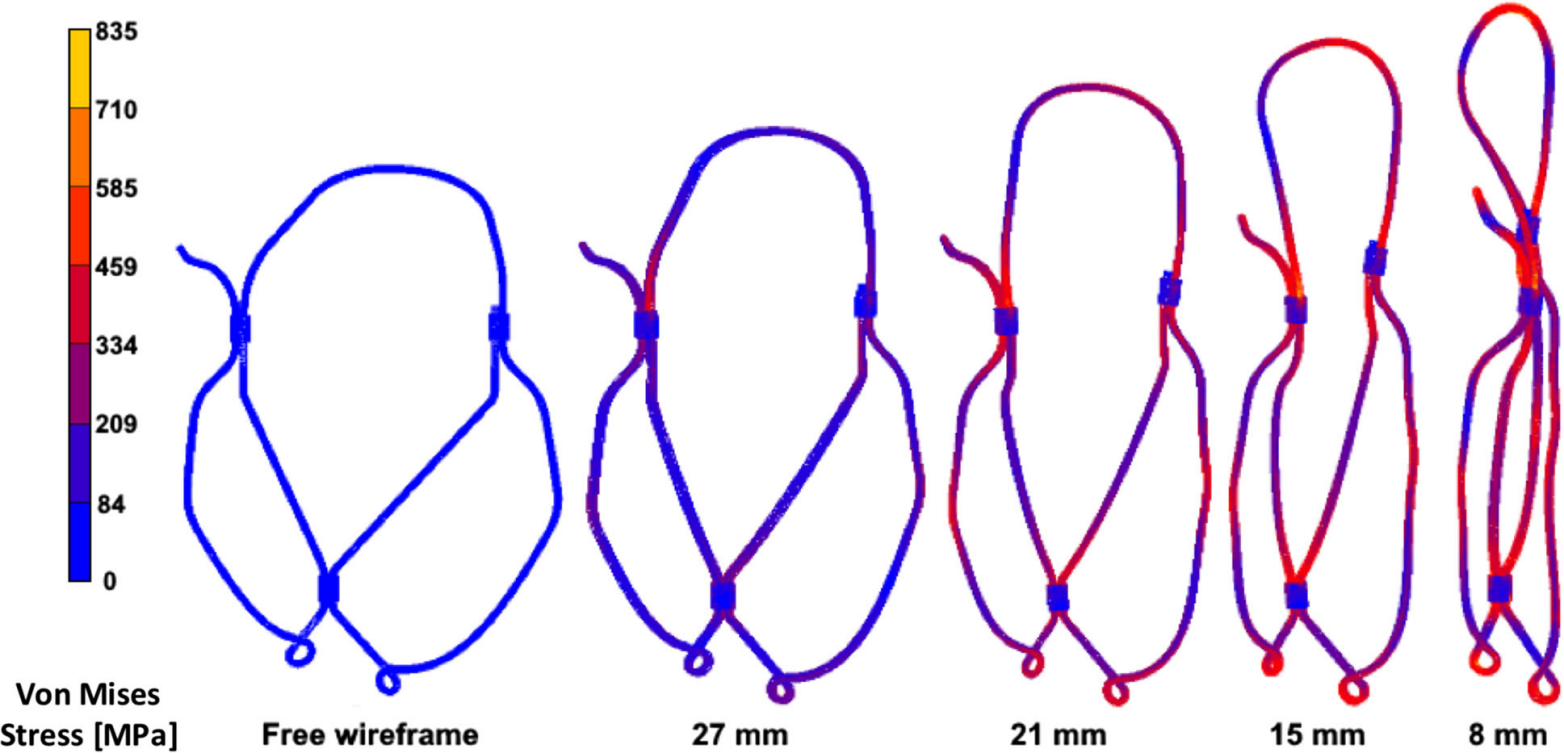
Stress distributions for the optimal geometry of the transcatheter mitral valve wireframe, crimped to different diameter sizes.

The selected design, characterised by a coaptation area of 1.8 cm^2^, met the peak maximum principal stress design criteria, with an estimated peak value below 5 MPa (3.51 MPa), located at the arris between the leaflets. The resulting stress distributions for the optimal geometry of the crimped wireframe are shown in Fig. 5. The critical points of maximum stress during crimping occurred around the sleeves. The highest stress, as expected, occurred at the maximum collapse diameter of 8 mm, and was 835 N/mm^2^. This remains below the yield stress reported for martensite in superelastic Nitinol, at the operating range of temperature.^46^

**Figure 6:**
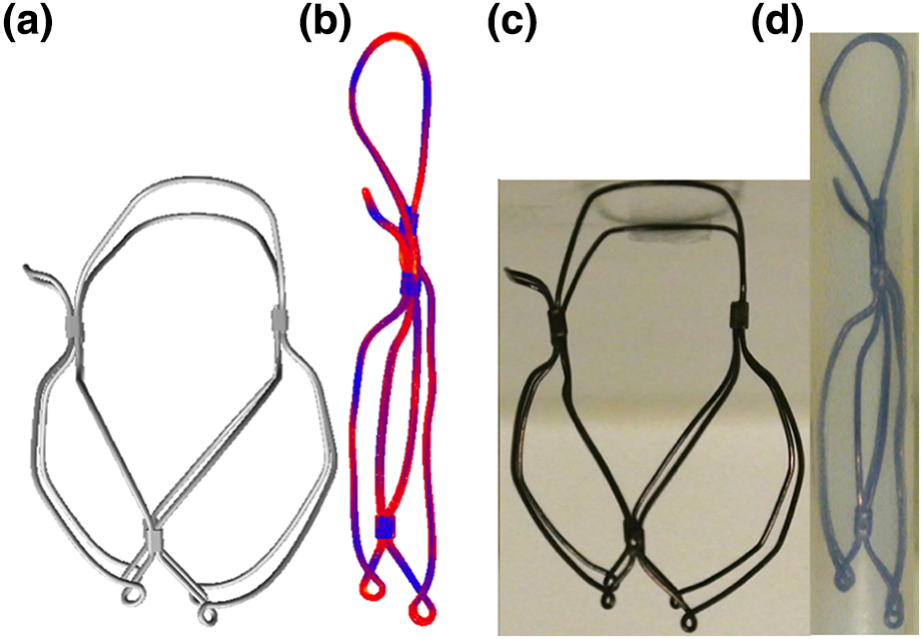
The transcatheter mitral valve wireframe: (a) solid model; (b) numerical model crimped in a 8 mm diameter cylinder; (c) manufactured prototype; (d) prototype crimped in a 8 mm diameter tube

The optimised wireframe geometry was closely replicated physically by thermomechanical processing of Nitinol wire, and mechanical crimping with stainless steel sleeves. Comparison between the free and crimped TMV wireframe geometries for the numerical model and prototype are given in Fig. 6.

Elastic deformation of the wireframe in an 8 mm diameter tube shows that the portions functioning as springs (Fig. 2a: portions 3 and 4) and the portions holding the mitral valve leaflets (Fig. 2a: portions 5 and 6) do not intersect with each other, this leaves sufficient space for the leaflets and sealing cuff when crimped. Additionally, the geometry of the crimped wireframe was in good agreement with the numerical prediction.

**Figure 7:**
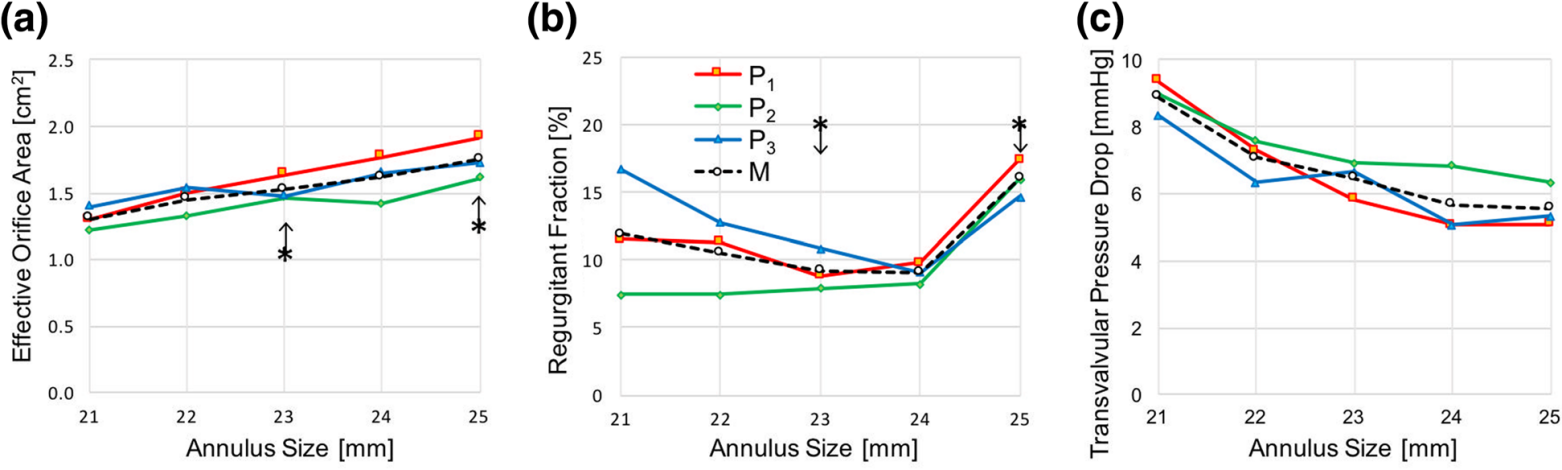
Hydrodynamic assessment results for the three tested prototypes (P_1_, P_2_, and P_3_; M represents the mean of the three tests) in six different annulus sizes: (a) effective orifice area; (b) regurgitation fraction; and (c) mean transmitral pressure difference during diastole. Minimum performance requirements for 23 and 25 mm, as per ISO 5840-3:2013, are indicated by the asterisk symbol, with the arrows pointing the allowed region.

Diagrams of the effective orifice area, regurgitant fraction and mean diastolic transmitral pressure difference for the prototypes in the different annulus sizes are represented in Fig. 7. The estimated EOA increased with the size of the host valve, with the mean for the three prototypes raising from 1.26 to 1.70 cm^2^ when moving from the 21 to the 25 mm annulus. All valves exceeded the effective orifice area required by the ISO 5840-3:2013 standard, for the different implantation sizes (larger than 1.05 cm^2^ and 1.25 cm^2^ for mitral annuluses of size 23 and 25 mm, respectively).

Regurgitant fractions did not show a clear pattern with the implantation size, and ranged from 8.2 to 17.8%. However, all prototypes met the minimum performance requirements in the ISO5840-3:2013 standard (regurgitant flow fraction ≤20% for both 23 and 25 mm annuli—no specifications for smaller sizes). The mean diastolic transmitral pressure difference decreased in the larger annuluses and reached a maximum value of about 9 mmHg in the 21 mm annulus, reducing to 5 mmHg in the 25 mm annulus.

**Figure 8:**
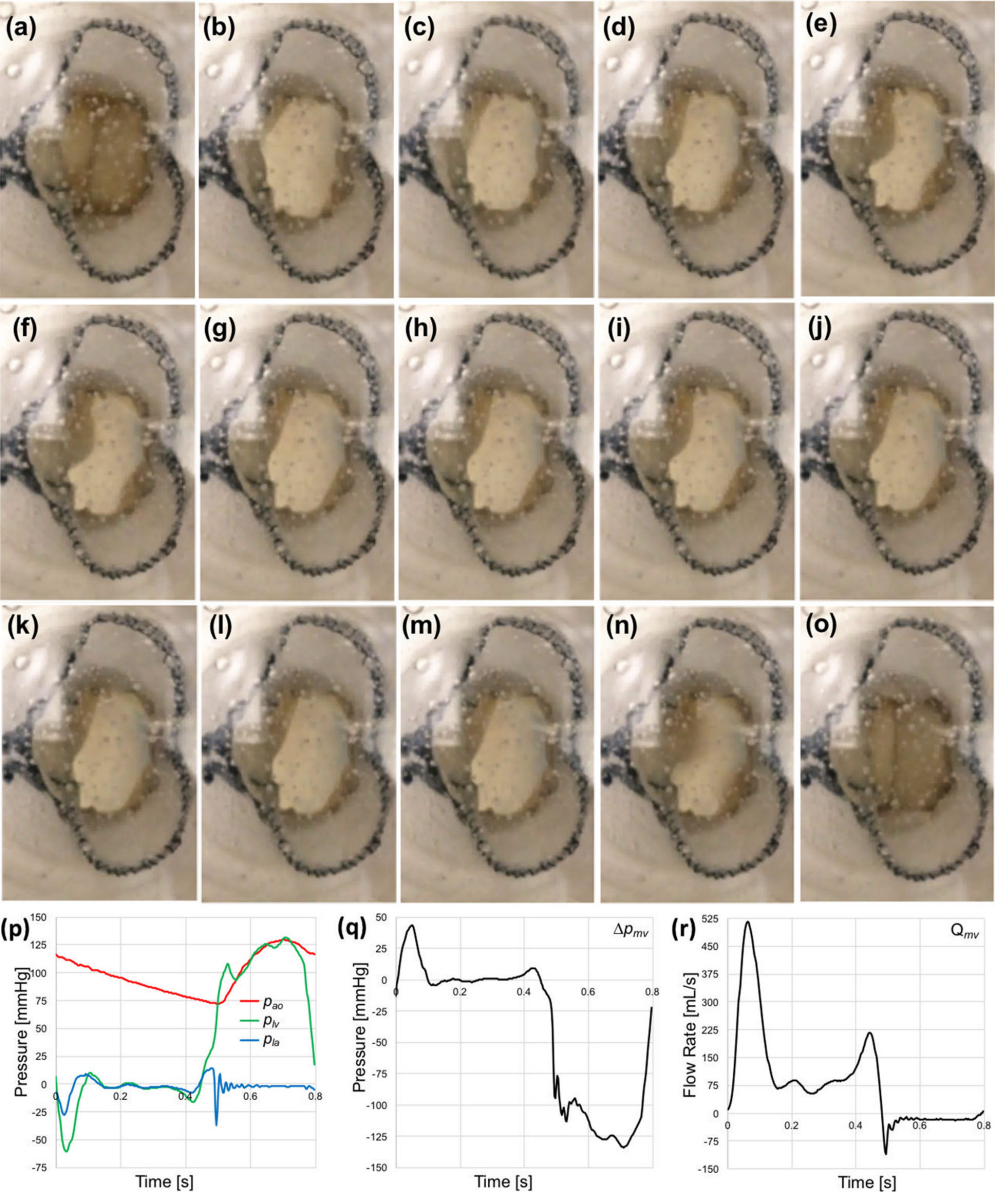
Sequence of snapshot images of one of the tested prototypes during the forward mitral valve flow for 23 mm implantation (a–o). The anterior and posterior leaflets are on the left and right side, respectively. For the test in the sequence are also reported: (p) left ventricular, left atrial and aortic pressure signals (p_lv_, p_la_ and p_ao_, respectively); (q) transmitral pressure difference signal (Δ*p*_mv_); and (r) flow rate signal through the TMV (*Q*_mv_)

A sequence of snapshot images of one of the prototypes acquired during the forward mitral valve flow for 23 mm implantation size with 29 fps frame rate are shown in Fig. 8a. The valve leaflets fully opened at the beginning of the left ventricular filling. The anterior leaflet remained fully open during the forward mitral valve flow while the posterior leaflet was fluttering. Duration of the leaflet open phase was approximately 60% of the entire cardiac cycle.

The peak (systolic) transmitral pressure difference was 125 mmHg, while the maximum diastolic opening pressure was about 45 mmHg. Regurgitant flow was observed over the ventricular systole, primarily due to paravalvular leakage between the mitral annulus and the device. The closing regurgitation (due to closure of the mitral valve leaflets) was higher in the larger annuluses. Anchoring was adequate for all tests, and no valve migration was observed for any of the test conditions. Typical pressure and flow rate diagrams through the valve, obtained for one of the three prototypes in an annulus of 23 mm over a cardiac cycle, are provided in Fig. 8.

## Discussion

Currently, no device specifically designed for TMV implantation has been approved for the European or American market. However, a number of solutions have been proposed, with many already at the stage of clinical trial (these include the *CardiAQ* 51,52 and *Fortis*,^2^,^8^ Edwards Lifescience; the *Tendyne*,^39^ Tendyne Holdings Inc., Roseville MN, USA; the *Tiara*,^14^ Neovasc, Richmond, Canada; the *NaviGate*, NaviGate Cardiac Structures Inc., Lake Forest, CA, USA; and the *Intrepid*, Medtronic, Dublin, Ireland).^31^ Despite the reduced number of patients involved in the trials and the large 30 days mortality rate, justified by the compassionate ground of the implants, this early experience has confirmed the potential benefit of the treatment and the ability of transcatheter solutions to successfully replace the mitral valve function.^31^ All devices under investigation are based on three occluding leaflet, replicating the configuration and function of semilunar valves. These are supported by self-expanding stents, obtained from laser-cut nitinol tubes, mechanically deformed and thermoset.^41^ The stents bulge or expand in a flange covered with a fabric material, designed to apply pressure on the atrial inflow portion, and used to minimise paravalvular leakage while counteracting the ventricular anchoring force providing the valve securing. From a technical point of view, a major distinction between the devices currently under investigation is represented by the method they use to generate the ventricular anchoring force, which can be based on ventricular tethers (e.g. *Tendyne*), native valve anchors (e.g. *CardiAQ*, *Fortis*, *Tiara* and *NaviGate*) or dual stent structures with barbs.^38^

The device presented in this paper introduces a number novel concepts, providing new and alternative features. Contrary to competing TMVs, the proposed solution is based on two asymmetric flexible leaflets, describing a D-shape cross section designed to better conform to the irregular anatomy of the valve annulus and minimise the disturbance to the sub-valvular apparati. This allows to maximise the geometrical orifice area of the prosthesis without interfering with the aortic valve anatomy and function. The leaflets are sutured onto a self-expanding frame, obtained from a nitinol wire, thermo-mechanically formed and mechanically crimped at five locations. This defines a set of arched ribs expanding into the atrium and two petal-like structures protruding into the ventricle between the native mitral leaflets, whose counteracting action generates the anchoring force, whilst limiting the systolic motion of the native anterior leaflet and the associated risk of left ventricular outflow tract obstruction. The wireframe configuration results in minimum metallic material, and relies on a skirt made from polymeric mesh (allowing integration from the host tissues), tensed between the atrial petals and the leaflets, to gently distribute the contact pressure over the annulus region. Paravalvular sealing is provided by a pericardial cuff extending around the entire framework of the valve, which inflates during systole as effect of the transvalvular closing pressure. The valve, designed in the presented version for transapical implantation, can be retrieved into the delivery system after complete expansion, using a similar mechanism to that described by the authors for a TAVI device.^44^

The structural numerical analyses, though inherently limited in their ability to represent the physics involved in heart valve leaflet closure, were adequate to predict the systolic function of the leaflets. In particular, this approximation does not take into account the interaction between the working fluid and the structural components, which determine the flow patterns and the pressure differences acting under real physiological conditions. Fluid structure interaction modelling would be more accurate for the simulation of the opening and closing leaflets dynamics. However, the peak of stress in the leaflets during the cardiac cycle is essentially led by the closing transvalvular pressure load,^33^ so that neglecting the local pressure variation and fluid shear stresses due to blood flow can still yield to sufficiently accurate results for the design evaluation stage.^10^

The valve wireframe optimisation was carried out until obtaining an optimal geometry which has lower stresses than NiTi yielding. Portions 5 and 6 in Fig. 2a were imposed by the leaflets geometry and kept unchanged for all wireframe models. The geometry of the wireframe is relatively complex, and includes a number of geometric parameters which needed to be optimised to obtain a suitable design. Each section was iteratively modified to minimise local stresses, resulting in a final geometry which fits adequately into the host mitral anatomy, maintaining acceptable levels of stress in the crimped configuration. The finite element analyses of a wireframe crimped to a diameter of 8 mm resulted in a maximum stress less than 900 MPa, which corresponds to a typical yield stress for Nitinol.^46^ The stress concentrations were predicted in the vicinity of the crimping sleeves, with local maxima around 600 MPa. Therefore, plastic deformation is not expected in the crimped wireframe, and this was confirmed by loading and unloading the physical prototype in an 8 mm diameter tube multiple times, without observable changes in shape. Besides, the presented version of the wireframe is designed to be ideally implantable from transapical route, which tolerates the use of larger sheath profiles (up to 33 French, 11 mm), resulting in further reduction of the stresses on the NiTi wireframe.^60^ Crimping of the TMV wireframe was simulated by gradually shrinking a cylindrical contact surface surrounding the prosthesis along its entire length. In the current application, the valve distal loops (Fig. 2a, portions 1 and 2) are engaged by a set of tethers, used to pull the valve into the catheter from the side at the outflow.^45^ Nevertheless, the resulting geometry of the crimped wireframe in the numerical simulations resulted visually accurate.

The valve design and prototypes were of a nominal size equal to 26 mm, corresponding to the largest inter-trigonal dimension of the prosthetic leaflets. This is suitable for patient’s annuli with inter-trigonal diameters equal or lower than 25 mm. Though this range is smaller than the average size in adult humans, it is more suitable for preclinical *in vivo* evaluation in ovine models,^43^ which is expected to be one of the next developmental steps. The prototypes were tested in mock host annuli of inter-trigonal diameters ranging from 21 to 25 mm. As expected, the diastolic transmitral pressure difference raised nonlinearly as the dimensions of the host annulus reduced, increasing from about 5 mmHg for the 25 mm annulus, to about 9 mmHg for the 21 mm annulus. A high peak in the initial diastolic transmitral pressure drop is measured in the tests (up to 45 mmHg). This is often observed in tests performed on hydro-mechanical pulse duplicators,^16^,^28^,^29^,^48^,^53^,^55^ and could be due to the non-physiological ventricular compliance, which may determine steeper flow waves and higher pressure gradients associated with early passive filling during ventricular relaxation. The calculated EOA well reflected the variation in the area of the implantation annulus, varying proportionally. Regurgitant fraction did not show a clear pattern associated with the implantation size for the different prototypes, although the mean value reduced progressively from 21 to 24 mm, inverting the trend at 25 mm. The reduction with the size may be associated with the different length of the mock native leaflets, which were designed proportional to the annulus size and, therefore, provided different covering of the sealing cuff of the prosthetic valves. On the other hand, the increased regurgitant fraction in the 25 mm annulus may be justified by the presence of gaps between the device and the mitral annulus. Globally, the device met the hydrodynamic requirements requested for transcatheter mitral valves in the standard ISO5840-3:2013, for all implantation sizes. Direct comparison of the hydrodynamic performance with competing solutions is not possible, as these are not available in the market and no *in vitro* data quantifying their diastolic and systolic efficiency have been published. However, measured values of transmitral diastolic pressure drops are consistent with those reported for transcatheter mitral implantation of off-label TAVI devices in failed mitral valve bioprostheses or annuloplasty rings, and in severe calcific mitral stenosis.^13^,^18^ Regurgitant fractions were inferior to those previously measured on the same system for commercially available TAVI devices.^45^ This is very encouraging, in consideration of the fact that, for the mitral position, closure is associated with higher transvalvular pressure drop and longer durations with respect to the cardiac cycle.

In terms of anchoring, no migration was observed for any of the test configurations, covering host annuli with inter-trigonal diameters between 21 and 25 mm. However, it needs to be taken into account that the mock host valves did not model the physiological contraction, and *cordae tendineae* and papillary muscles were absent. *Ex vivo* isolated beating heart or pressurised animal heart platforms^17^,^57^ and acute in animal trials could provide more reliable insights on the fitting and performance of a transcatheter valve.^44^ These studies would also be essential to verify the efficacy of the anchoring mechanism to avoid left ventricular outflow tract obstruction by preventing the systolic motion of the native anterior leaflet.

## Conclusion

A novel TMV was developed, consisting of two bovine pericardial leaflets designed to ensure proper functionality across a range of implantation configurations and a sealing cuff, supported by a wireframe, optimised to minimise stresses whilst crimped. The device exceeded the minimum performance requirement from the international standards, thereby proving its feasibility as a mitral valve substitute to treat mitral regurgitation. *In vitro* durability assessment of the valve by means of accelerated cyclic tests is now being conducted, with the aim of verifying that the solution guarantees a survival equal or superior to the requirement for flexible leaflets heart valves (200 × 10^6^ cycles). The next steps in the development will include *in vivo* preclinical evaluation by means of in animal implants (possibly complemented by *ex vivo* studies), to validate the design principles and the efficacy of the device.

If these will confirm the predicted performance, the proposed device could provide a viable alternative to transcatheter repair techniques and, due to its geometric similarity to the human mitral valve anatomy, may result a more appropriate option compared to the other TMVs in development.

## Electronic supplementary material

Below is the link to the electronic supplementary material.

Supplementary material 1 (MOV 21234 kb)

Supplementary material 2 (MOV 20581 kb)
